# Harnessing cancer stem cell-derived exosomes to improve cancer therapy

**DOI:** 10.1186/s13046-023-02717-x

**Published:** 2023-05-23

**Authors:** Jianqiang Yang, Yong Teng

**Affiliations:** 1 Department of Hematology and Medical Oncology, Winship Cancer Institute, Emory University School of Medicine, 201 Dowman Dr, Atlanta, GA 30322 USA; 2 Wallace H. Coulter Department of Biomedical Engineering, Georgia Institute of Technology & Emory University, Atlanta, GA 30322 USA

**Keywords:** Cancer stem cells, Exosomes, The tumor microenvironment, Therapeutic target, Cancer anticancer strategy

## Abstract

Cancer stem cells (CSCs) are the key “seeds” for tumor initiation and development, metastasis, and recurrence. Because of the function of CSCs in tumor development and progression, research in this field has intensified and CSCs are viewed as a new therapeutic target. Exosomes carrying a wide range of DNA, RNA, lipids, metabolites, and cytosolic and cell-surface proteins are released outside of the originating cells through the fusion of multivesicular endosomes or multivesicular bodies with the plasma membrane. It has become evident that CSC‐derived exosomes play a significant role in almost all “hallmarks” of cancer. For example, exosomes from CSCs can maintain a steady state of self-renewal in the tumor microenvironment and regulate microenvironmental cells or distant cells to help cancer cells escape immune surveillance and induce immune tolerance. However, the function and therapeutic value of CSC‐derived exosomes and the underlying molecular mechanisms are still largely undefined. To provide an overview of the possible role of CSC‐derived exosomes and targeting strategies, we summarize relevant research progress, highlight the potential impact of detecting or targeting CSC‐derived exosomes on cancer treatment, and discuss opportunities and challenges based on our experience and insights in this research area. A more thorough understanding of the characteristics and function of CSC‐derived exosomes may open new avenues to the development of new clinical diagnostic/prognostic tools and therapies to prevent tumor resistance and relapse.

## Background

Cancer remains one of the leading causes of death globally. Whether in developed or developing countries, men, or women, multiple tumor types present major risk factors endangering human life and health. In 2019, there were an estimated approximately 23.6 million new cancer cases and 10.0 million cancer deaths globally [1]. In past years, there have been great advances in cancer treatment, including surgery, chemotherapy, radiotherapy, targeted therapy, etc. Although the incidence and mortality of most cancer types have declined [2], improving the therapeutic effect of cancer treatments remains a challenge for clinicians and scientists. Recurrence, metastasis, and treatment resistance are three important reasons for poor treatment outcomes [3], and can be explained at least partially by the presence of cancer stem cells (CSCs) [4, 5]. Accumulating studies have shown that CSCs are closely related to recurrence, metastasis, heterogeneity, and resistance through their ability to arrest in the G0 phase and rapidly generate large numbers of new heterogeneous cancer cells [6]. It is of great significance to regard CSCs as a new therapeutic target. Understanding how CSCs communicate with surrounding cells and distant organs is a prerequisite for clarifying the specific mechanism by which CSCs trigger tumor initiation and development. The role of exosomes as carriers of this type of delivery is critical. Most of the current research focuses on cancer cell exosomes, but as an important “seed”, research on CSC-derived exosomes is still limited. Given that exosomes carry genetic material from parental cells, it is reasonable to believe that CSC-derived exosomes also have a specific function in cancer progression. This article will review the function of CSC-derived exosomes and provide a perspective on related research progress.

### Characteristics, role and clinical implications of CSCs

The CSC hypothesis was first proposed by Mackillop in 1983 and CSCs were identified in leukemia and subsequently isolated in 1997 by surface labeling expression of CD34^+^ and CD38^−^ [7]. Whether CSCs represent a distinct cell type or a cell state within the tumor remains a matter of debate in the scientific community. While both models may be correct to some extent, recent studies suggest that CSCs possess unique stem cell-like properties and express distinct markers, suggesting that they represent a distinct cell type. However, CSCs can also arise from other cancer cells under certain conditions, suggesting that they may represent a phenotypic state.

Different surface markers may be expressed in different cancers, such as ALDH^+^, CD44^+^, CD133^+^ expressed in head and neck cancer [8] and CD200^+^, CD166^+^ expressed in colorectal cancer [9, 10]. According to these markers, specific separation of CSCs can be carried out. In addition to maintaining self-renewal, CSCs have “plasticity,” which allows them to differentiate into subtype cancer cells under certain conditions, such as changes in the peripheral environment and regulation by various immune factors (Table 1) [11–13]. This phenomenon is subject to certain spatiotemporal characteristics and will produce cancer cell subtypes adapted to the environment with different external stimuli, thus providing a reliable seed for continuation. In contrast to normal adult stem cells, the differentiation of CSCs is disorderly, for the most part, uncontrolled, and it is for this reason that tumors develop [14]. CSCs are thought to be responsible not only for tumor growth, maintenance, and resistance to chemotherapy and radiotherapy, but also to be involved in cancer recurrence after treatment as they have the ability to regenerate the tumor (Table 1). Non-CSCs are thought to be more differentiated and less likely to drive tumor growth or recurrence. In addition, CSCs can evade the immune system through various mechanisms, such as downregulating the expression of surface antigens that would normally trigger an immune response or secreting factors that suppress immune cell activity. This allows CSCs to persist and proliferate even in the presence of an active immune system. Interestingly, CSCs share some conserved signaling pathways with normal adult stem cells, including Wnt/β-catenin, Notch, PI3K/AKT/mTOR pathways, etc., and these signaling pathways also can be used as targets for CSCs. However, CSCs often exhibit a dysregulated regulatory mechanism compared to normal adult stem cells. While adult stem cells are tightly regulated by feedback mechanisms that ensure proper differentiation and self-renewal, CSCs can exhibit uncontrolled self-renewal and differentiation, leading to tumor formation and proliferation.

**Table 1:** Distinct characteristics between CSCs and non-CSCs

CSCs	Non-CSCs
Small amount (0.01–2%), rare within tumors most of the time, but can increase to more than 30% in short time [15]	Large quantity, almost majority within tumors
Self-renewal, multipotency, and symmetric/asymmetric divisions	Limited proliferation and symmetric divisions
Reside predominantly in hypoxic, low pH and low nutrient niches [16]	Stochastic distribution
Dysregulated cell cycle, reversible cellular quiescence capability [17]	None
High tumorigenic capacity	low
Ability to induce therapeutic resistance [18]	The main target of treatment until the emergence of unresponsive cancer cells
Highly responsive to changes in the tumor microenvironment	low
High heterogeneity than non-CSCs counterpart in tumors	Low heterogeneity
Ability to arrange a hierarchy of daughter cells [19]	None

The proportion of CSCs in tumor tissues is very low and generally accounts for only 0.01–2% of the total tumor mass (Table 1). Therefore, it is very challenging to isolate CSCs. There are still some reliable isolation techniques that have been widely used. At present, the main separation methods are as follows: through the identification and combination of surface markers, separation is carried out by fluorescence-activated cell sorting (FACS) and magnetic-activated cell sorting (MACS). FACS is widely used since it can be used for sorting multiple biomarkers at one time and has strong specificity. MACS is simpler but the technology is more complex. Both methods require a large number of cells to be isolated. Moreover, CSCs can be isolated as side population (SP) cells since they resist the nuclear dye Hoechst 33,342. ABCG2 is highly expressed in SP cells, and studies have reported that ABCG2 is highly correlated with drug resistance, which indicates that it can be used as a marker of high resistance CSCs [20]. There is also a serum-free medium (SFM) sorting method, which utilizes the characteristic that CSCs can grow in a spherical suspension in SFM. By adding specific growth factors and additives, after several generations of culture and proliferation, more CSC spheres can be obtained. These factors and additives include EGF, bFGF, heparin, etc. Researchers are increasingly using more than two methods for extraction to obtain a larger number of CSCs of higher purity.

The properties of CSCs may allow the application of CSC-based approaches to develop personalized treatments tailored to a patient's particular cancer type and genetic profile. Through isolating CSCs of primary tumors, CSC models can be used to identify predictive biomarkers for cancer prognosis and evaluate the effectiveness of therapeutic approaches. By identifying different markers, the origin of tumors can be determined, which provides an essential supplement for diagnosing unknown tumors. However, the relevant technologies are not yet mature due to the small number of CSCs typically isolated and the need to improve cultivation methods.

### Exosomes and CSC-derived exosomes

Exosomes were first identified in sheep reticulocytes in 1981 [21] and were at first thought to be a “trash” compartment containing discarded cellular components. In 2007, Valadi et al. found that cells can exchange genetic material through exosomes and even transmit information to tissues that are far away [22]. Exosomes are nano-sized vesicles (about 30–150 nm) that have a lipid bilayer and carry various biomolecules, including proteins, glycans, lipids, metabolites, RNA, and DNA. They are secreted by almost all cells. In addition to exosomes, cells also produce other types of extracellular vesicles (EVs), including microvesicles (MVs), formed by direct plasma membrane budding, which are thought to be larger than exosomes, ranging in size from 100 to 1000 nm, apoptotic bodies and oncosomes.

The mechanism of exosome formation is still not very clear, but the process may be similar in different types of cells. Exosome biogenesis involves double invagination of the plasma membrane and the formation of intracellular multivesicular bodies (MVBs) containing intraluminal vesicles (ILVs) through MVBs and cell membrane fusion and exocytosis. The first invagination of the plasma membrane forms a cup-shaped structure containing membrane surface proteins and extracellular soluble proteins, which form the early sorting endosome (ESE), and in some cases early endosomes may directly merge with pre-existing early endosomes and become late endosomes through the mediation of the endoplasmic reticulum and Golgi complex. The endosomal sorting complexes required for transport (ESCRT) function in a certain order in this process. As the number of multi-luminal vesicles gradually accumulates in endosomes, MVBs are formed, which can either fuse with lysosomes or autophagosomes and be degraded or be pulled by intracellular molecular motors. MVBs fuse with the cell surface and secrete multi-luminal vesicles (including exosomes) outside the cell. Exosomes are taken up by other cells, and their cargoes are transferred and influence the recipient cells. As such, exosomes are appreciated to be essential mediators of cell–cell communication.

**Fig. 1:**
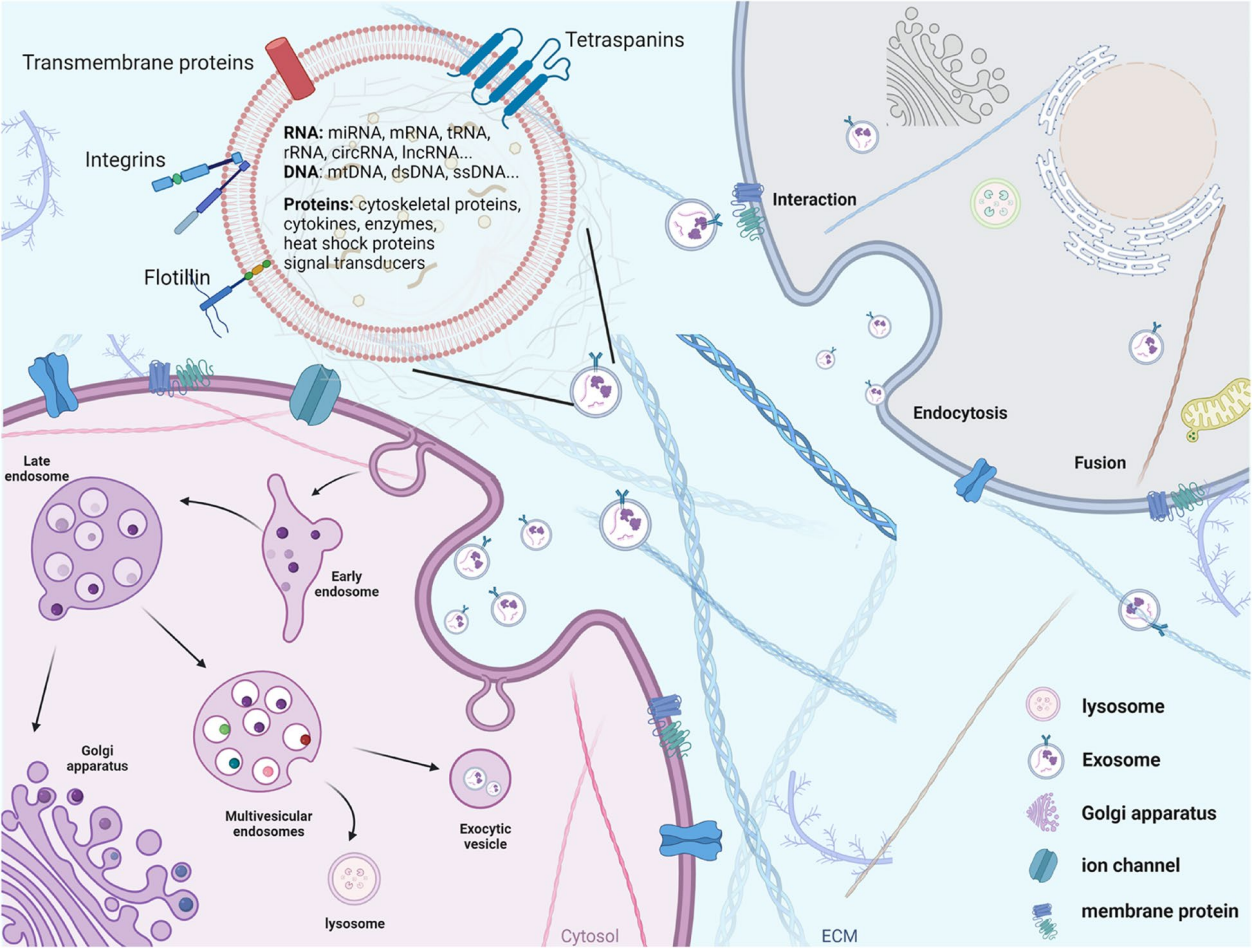
Exosome biogenesis and internalization mechanisms. Exosomes are surrounded by a phospholipid bilayer comprising various cell surface proteins (including tetraspanins, flotillins, integrins and transmembrane proteins) that mediate the orientation and connection of exosomes. Exosomes carry various biological species, including intracellular proteins, nucleic acids (including DNA and RNA) lipids and metabolites, and regulate the function of target cells by releasing their cargo. Exosome biogenesis begins from the double invagination of the plasma membrane, forming an early endosome and subsequently mature to late endosome. Then, multivesicular bodies (MVBs) form, which contain intraluminal vesicles (ILVs). MVBs can be fated for lysosomal degradation or fusion with the plasma membrane as exocytic vesicle to release exosomes. Exosomes have mainly three ways to communicate with cells: (1) interact with protein or receptor on the membrane, (2) exosome membrane fuses with the cell membrane, and the contents of the exosome are directly delivered into the target cell, and (3) exosomes enter cells through phagocytosis or endocytosis

At present, three mechanisms of signal transduction between exosomes and recipient cells have been identified. Firstly, transmembrane proteins on exosomes directly act on signaling molecules on the surface of receptor cell membranes to activate intracellular signaling cascades. Secondly, the exosome membrane fuses with the cell membrane, and the contents of the exosome are directly delivered into the recipient cell. Thirdly, exosomes enter cells through phagocytosis or endocytosis (Fig. 1). Exosomes can reflect the composition of the source cells, but, importantly, exosomes secreted by the same cell in different states are not consistent, and thus exosomes reflect the physiological status of cells under different conditions. For example, in a hypoxic environment, exosomes secreted by cancer cells are rich in a variety of hypoxia-regulated RNA and proteins. Recent studies indicate a functional, targeted, and mechanistically driven accumulation of specific cellular components in exosomes, suggesting that they play a more important role in regulating intercellular communication [23]. Cancer cells can release corresponding exosomes to find target organs and connect tumors with the microenvironment of target organs. Importantly, CSC-derived exosomes have been shown to contain higher levels of stemness markers and proteins, such as CD133, CD44 and Notch1, which can be transferred to non-CSCs to enhance their stemness. In addition, CSC-derived exosomes can induce dynamic tumor heterogeneity in the tumor microenvironment (TME) by delivering specific proteins and transcription factors compared to non-CSC-derived exosomes. Exosomes transfer information by releasing the luminal cargo of proteins, nucleic acids (DNA and RNA), lipids and metabolites (Fig. 1). Lipids form the bilayer of the exosome membrane, maintaining stability of the structure, which is critical for the release of exosomal cargo. Exosomal proteins comprise surface and intracellular proteins. Surface proteins involved in the formation and release of exosomes include the tetraspanins family (CD9, CD63, CD81 and CD82), flotillin, integrins and transmembrane proteins. Intracellular proteins include cytoskeletal proteins, cytokines, heat shock proteins and enzymes (Fig. 1). There are many nucleic acids in exosomes, including mRNA, tRNA and ncNA (Fig. 1). miRNA (miRNAs) are the most abundant cargo molecules in exosomes and can be functionally delivered to target cells.

Recent research has focused on the role of exosomes secreted from CSCs in modulating CSC niches. Characteristics of CSC-derived exosomes are distinct from those of non-CSC-derived exosomes (Table 2). It has been reported that CSCs release various cytokines and factors, including IL-6, TGF-β, CD44, and ALDH, to regulate immunomodulatory function, stemness and differentiation among CSCs and the TME. For example, when breast CSCs were co-implanted with breast cancer cells, metastasis increased, and DKK1 was identified as a key factor secreted by breast CSCs that mediates these functions [24]. Nevertheless, few reports have investigated the functional properties of CSC-derived exosomes. Given the importance of CSCs, it is reasonable to believe that CSC-derived exosomes are essential in the communication among CSCs and other microenvironmental cells.

**Table 2:** Distinct characteristics of CSC-derived exosomes vs non-CSC-derived exosomes

Exosomes derived from CSCs	Exosomes derived from non-CSCs
Specific stem cell markers (e.g., CD133, CD44 and CD200) and stem cell-related cargoes	None
Involvement in the initiation of tumorigenesis by altering the tumor microenvironment	None
Involvement in tumor angiogenesis formation [25]	Low
Involvement in the tumor immunosuppressive microenvironment [26]	Low
Involvement in multiple signaling pathways to regulate treatment resistance	Low
Critical for establishing premetastatic niche and colonizing metastatic site [27]	Low

### Role of CSC-derived exosomes in cancer progression

#### Exosomes maintain the stemness of CSCs

The molecular crosstalk between CSCs and their microenvironment plays an important role in maintaining their stem cell phenotype and function, which is the so-called CSC niche. The CSC niche consists of cell types such as cancer cells, immune cells, endothelial cells, mesenchymal stem cells (MSCs), and cancer associated fibroblasts (CAFs) as well as growth factors, chemokines, and cytokines [28]. For example, gastric cancer mesenchymal stem cells can enhance the properties of CSCs and tumorigenesis via PD-L1 signaling [29]. A study revealed that CD10^+^GPR77^+^ CAFs sustain CSCs stemness, which is driven by persistent NF-κB activation via p65 phosphorylation and acetylation [30]. Accumulating studies have shown that the crosstalk of CSC niches is delivered by exosomes [31–33]. Exosomes secreted by cancer cells can be taken up into CSCs involved in pathways related to tumor stemness, which further forms a positive promoting effect to maintain heterogeneity [34]. Studies have shown that Wnt, Notch and Hedgehog signaling pathways participate in the regulation of tumor stemness [35], and non-coding RNA (ncRNA) contained in exosomes can participate in the Wnt or Notch signaling pathway to regulate the spheroid forming ability of CSCs and the tumorigenic size of cancer cells [36–39]. Under hypoxia, exosomes from CAFs transfer circHIF1A into breast cancer cells, which absorb miRNA-580-5p by regulating the expression of the surface marker molecule CD44 of breast CSCs [40]. It was reported that cancer cells isolated from colon cancers re-express CSC markers after co-culture with fibroblasts and restored tumorigenicity, suggesting that the stemness of cancer cells is not immutable and can be regulated [41]. On the other hand, CSC-derived exosomes carry stem related factors such as OCT-4, SOX-2, and NANOG, or lncRNA/microRNA mediated with surrounding cells to enhance the expression of stemness [42, 43]. Studies have shown that some CSC markers also affect the exosome release, migration, and invasion ability of cancer cells [44, 45]. DCLK1 is also considered a CSC marker and has been shown to affect exosome biogenesis in a kinase-dependent manner by inducing increased exosome release and reprogramming of contents to facilitate the acquisition of a migratory phenotype [46, 47].

#### Exosomes maintain the transformation of CSCs and non-CSCs

As mentioned earlier, CSCs and non-CSCs are in a state of dynamic equilibrium in response to changes in the TME [48]. Similarly, exosomes are involved in the maintenance of this homeostasis [49]. Exosomal FMR1-AS1 secreted from esophageal carcinoma CSCs can activate TLR7-NFκB signaling and increase the expression level of c-Myc, which results in esophageal squamous cell carcinoma (ESCC) cell proliferation, anti-apoptosis, and invasion [50]. The transformation can be explained in two ways. First, the transformation of cell entities: CSCs can differentiate into cancer cells as the supplement in the pattern of asymmetric division. Cancer cells can be dedifferentiated and transformed into CSCs to maintain a stable number of CSCs. The reverse differentiation of cancer cells to an immature state under the influence of their TME represents the first step towards epithelial-mesenchymal transition (EMT) and the acquisition of stemness characteristics. EMT causes cancer cells to lose polarity and acquire a more invasive interstitial phenotype. EMT also promotes the transformation of epithelial non-CSCs into mesenchymal CSCs capable of generating cancer cells and promoting cancer cell proliferation, in which the transcription and proteomics of cell genesis are very complex. Second, and more complex, is the transformation of molecules: CSCs transmit regulatory signals and factors to the TME, playing a stimulatory role in the differentiation and division of other cells [51]. Studies have shown that CSC-derived exosomes can induce the generation of CAFs, which may be related to TGF-β [52].

#### CSC-derived exosomes promote neo-angiogenesis and metastasis

Angiogenesis is recognized as the essential ability of cancers to form blood vessels, which supply cancer cells with nutrition and oxygen that support their survival and growth. Many studies have shown that angiogenesis is involved in cancer growth and metastasis [53–55]. CSCs are considered to facilitate angiogenesis by dedifferentiating to endothelial cells as well as secreting proangiogenic and angiogenic factors, which are regarded as effective targets to block angiogenesis [56, 57]. Moreover, normal endothelial cells can activate angiogenic signaling pathways to stimulate new vessel formation by the uptake of cancer cell released exosomes. A study showed that CSC-derived EVs can transform normal fibroblasts into CAFs with enhanced oncogenic potential through up-regulating the β-catenin/mTOR/STAT3 pathway and increasing mRNA and protein levels of TGF-β1 [58]. Another study showed that the differentiation of endothelial precursor cells in angiogenesis can be traced back to CSCs. Exosomes facilitate cell communication by delivering miRNAs, which mediate an important aspect of the endothelial cell-to-cancer stem like cell crosstalk [59]. Many angiogenic factors such as VEGF, IL-8, and TNF-α are associated with CSCs in some types of cancer, and exosomes are still believed to be involved [25, 60–67]. Glioblastoma has a high level of miR-21 that upregulates VEGF expression. Sun and his colleagues found that exosomes released from glioma stem cells can promote the angiogenic ability of endothelial cells (ECs) by stimulating the miR-21/VEGF/VEGFR2 signaling pathway [60]. It has been shown that exosomes containing miR-155 secreted by gastric cancer cells significantly increase the rate of tumor angiogenesis by enhancing the expression of VEGF [65].

**Fig. 2:**
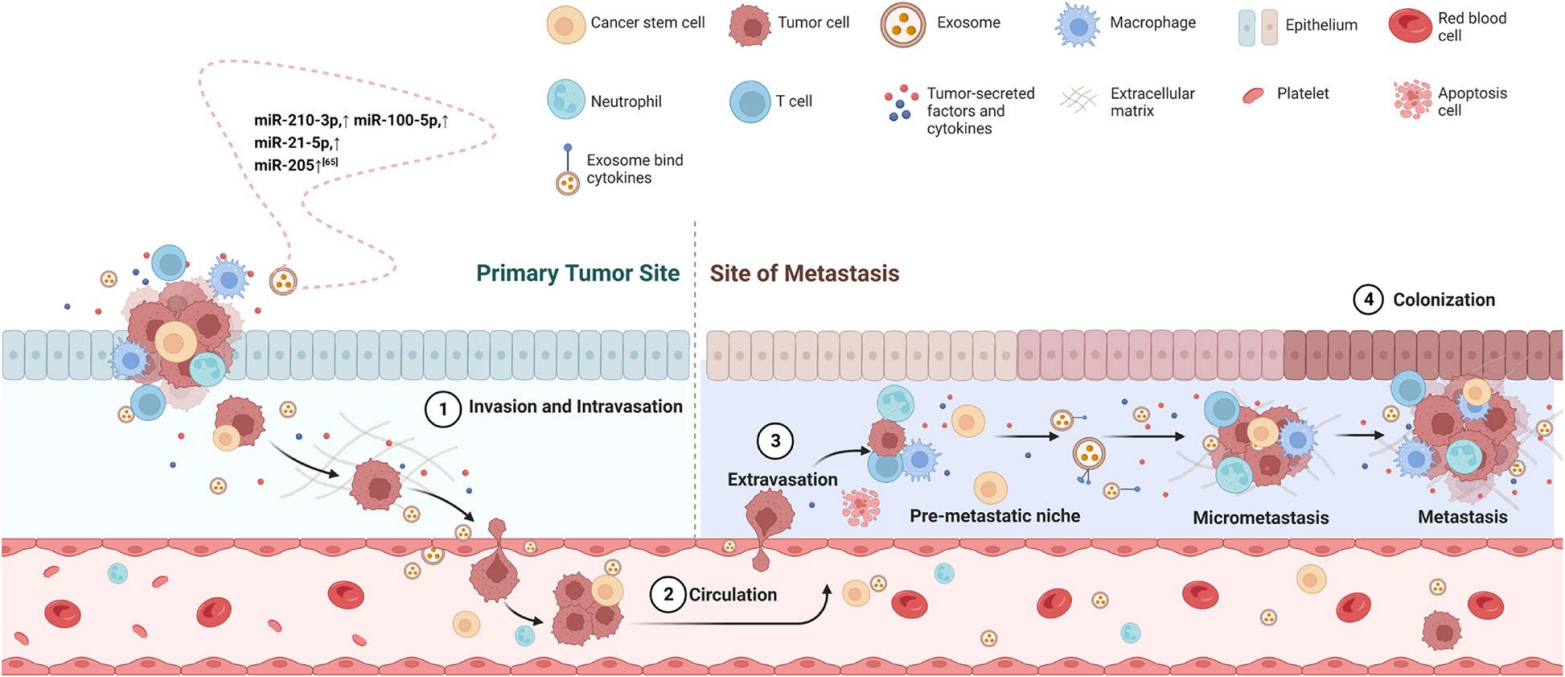
CSC-derived exosomes regulate the initial invasion and metastasis site of cancer cells. Establishment of distant metastasis may partially result from exosome-carried signals shared between adjacent cells and long-distance cells in the body. Step 1, invasion and intravasation: cancer cells in situ break through basement membrane with the infiltration of immune cells and enter the blood vessel through the endothelial cells. Exosomes regulate the surrounding cells to promote EMT and metastasis. Step 2, circulation: exosomes and cancer cells transport to the distant site with blood fluid. Step 3, extravasation: exosomes and cancer cells enter interstitial through the endothelial cells. Exosomes bind to specific tumor microenvironmental cytokines and are taken up in specific tissues, forming a pre-metastatic niche, which can recruit CSCs and cancer cells to reside. Step 4, colonization: exosomes and cancer cells colonize distant target organs to further facilitate neoplastic growth

CSCs are believed to be responsible for the development of metastasis, as they are more likely to survive in the process of metastasis and seed a new tumor in a different location (Fig. 2) [68]. EMT is an important step in metastasis, allowing cancer cells to detach from the primary tumor, migrate and invade other tissues. EMT has been implicated in carcinogenesis and confers metastatic properties, supporting the maintenance of CSCs by increasing their motility which enables these cells to move to more favorable environments in the body [69]. CSCs have been shown to promote EMT through ncRNA or proteins in several studies [70–75]. A study reported that cancer stem-like cells from thyroid cancer lines can transfer lncRNA-ROR to induce EMT and colonize the local TME and the distant metastatic niche [74]. Another study showed that exosomal lncRNA DOCK9-AS2 derived from PTC-CSCs can activate the Wnt/β-catenin pathway to aggravate stemness, proliferation, migration, and invasion in papillary thyroid carcinoma [75]. Resveratrol, an inhibitor of EMT, not only inhibits EMT and metastasis, but also suppresses stem cell-related markers and proliferation and induces cell apoptosis [76, 77]. A study from 2019 showed that CD103^+^ acted to guide CSC-derived exosomes to target cancer cells and organs, and these exosomes transported miR-19b-3p into clear cell renal cell carcinoma cells and initiated EMT promoting metastasis [78]. Since CSCs promote a pre-metastatic niche (PMN) over long distances, as an intercellular communication media, CSC-derived exosomes can transmit related signaling. A large volume of DNA, miRNAs, lncRNAs, lipids, proteins and other substances contained in exosomes reaches the recipient cell in the bloodstream or in a paracrine form to achieve long-distance or intercellular communication to promote tumor growth, metastasis, and drug resistance. Wang et al. found that lung CSC-derived exosomes promoted the migration and invasion of lung cancer cells, upregulated the expression levels of N-cadherin, vimentin, MMP-9 and MMP-1, and downregulated E-cadherin expression. Further, they verified that these exosomes contribute to a pro-metastatic phenotype in lung cancer cells via miR-210-3p transfer [27, 79]. Reduction of exosome secretion via depletion of Rab27a in cancer cells or pharmacological inhibition of exosomal uptake at sites of future metastases was sufficient to impair PMN formation and decrease spontaneous metastasis in tumor bearing mice [80, 81]. Indeed, CSC-derived exosomes could promote intensive research given their ability to regulate metastasis. In fact, angiogenesis is required for invasive tumor growth and metastasis [82–85]. A study revealed that ovarian cancer cell-secreted exosomal miR-205 promotes metastasis by inducing angiogenesis [86].

#### CSC-derived exosomes regulate autophagy of cancer cells

Autophagy is regarded as self-degradation processes that have pivotal roles in controlling the quality of cellular components and maintaining cellular homeostasis [87–90]. Over the past decades, the mechanisms by which autophagy contributes to stemness and why stem cells are more dependent on autophagy than non-stem cells have been intensively studied. Autophagy is a double-edged sword in cancer, playing a dual role as a tumor suppressor and promoter, and dysregulation of autophagy has been implicated in the development and progression of various types of cancer [91, 92]. The mechanisms underlying the regulation of autophagy by exosomes are not fully understood, but several signaling pathways have been implicated. For example, exosomes can transfer miRNAs to cancer cells, which can target autophagy-related genes and modulate autophagy [89]. Additionally, exosomes can activate various signaling pathways, such as the PI3K/Akt/mTOR pathway, which is a key regulator of autophagy [93, 94]. Autophagy is strongly associated with CSC maintenance and aggressiveness and is an adaptive mechanism of CSCs in the TME, which indicates that inhibition of autophagy may be a strategy for the treatment of metastasis. Autophagy allows CSCs to survive despite hypoxia and low levels of nutrients in the TME and is considered a major cause of survival and chemotherapy resistance in CSCs. Few studies have been reported on the relationship between autophagy and CSC-derived exosomes. Rotenone is a naturally occurring chemical compound that induces autophagic vacuolation in CSCs, and this process is associated with mitochondrial damage. Kumar and colleagues found that exosomes released from rotenone-treated prostate and breast CSCs expressed higher levels of exosomal markers (such as CD9, CD63, CD81, Alix and TSG101) compared to untreated CSCs [95]. This study supports the notion that autophagosomes in CSCs may associate with the protein complexes, ribosomes, endoplasmic reticulum and peroxisomes to form multivesicular endosomes that release exosomes. Nonetheless, it is timely to determine if and how CSC-derived exosomes regulate autophagy in cancer cells.

#### CSC-derived exosomes facilitate cancer cell immune evasion

**Fig. 3:**
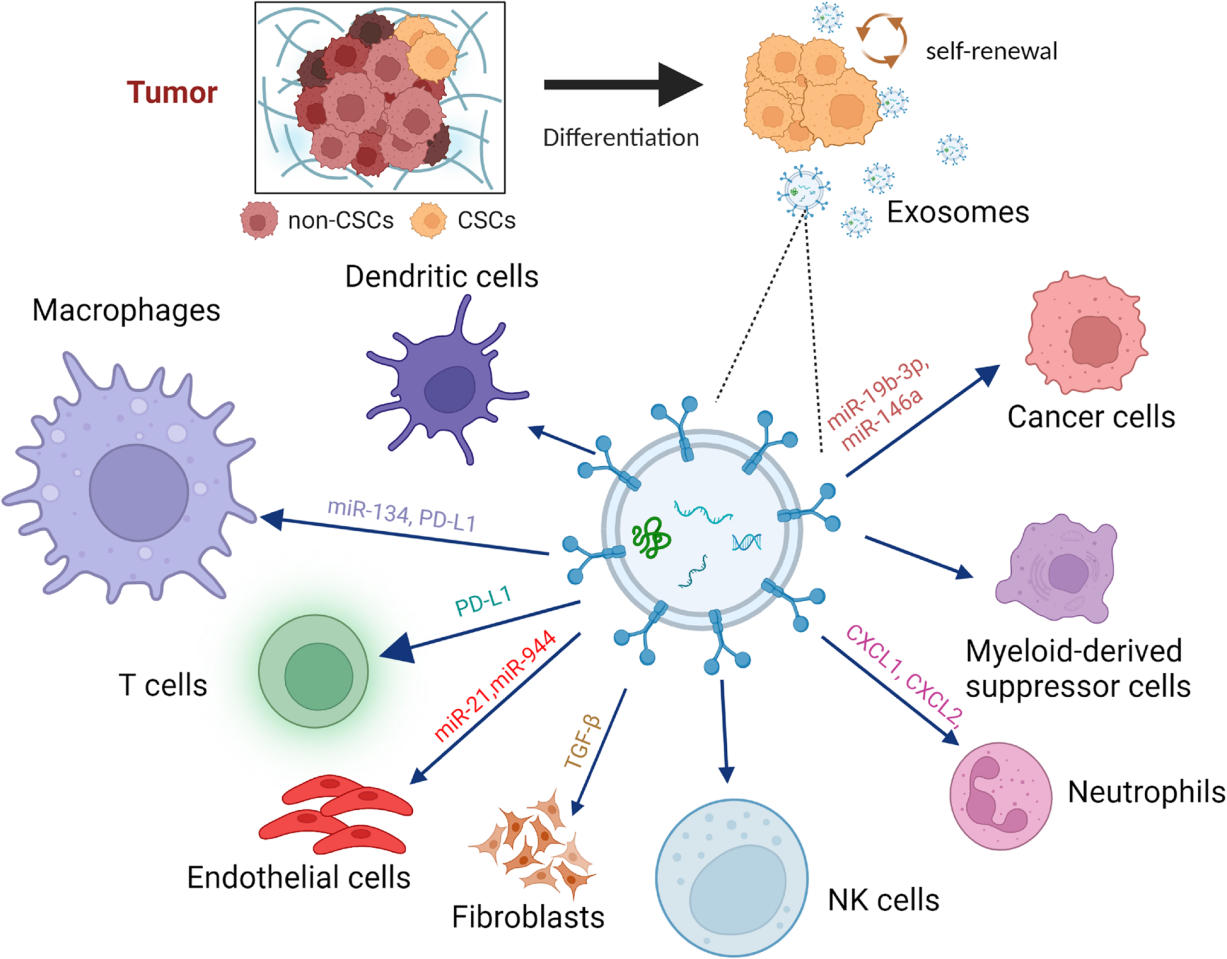
CSC-derived exosomes regulate surrounding stromal cells in the complex TME. CSCs can regulate other microenvironmental cells through releasing exosomes carrying CSC-specific DNA, RNA, lipids, metabolites, and cytosolic and cell-surface proteins, which enhance cancer cell proliferation, modify endothelial cells to promote vascular leakiness, and regulate T cells, macrophages, and neutrophils to suppress cancer immunity and activate CAFs to promote EMT [60, 102–106]

Immune cells actively monitor and eliminate cells that undergo malignant transformation. However, some of the transformed cells can evade immune surveillance and eventually form a tumor. CSCs play a central role in immune evasion, which is a hallmark of malignancy. The interaction between CSCs and tumor-infiltrating immune cells is complex and not fully understood. Some studies have suggested that CSCs can evade the immune system by downregulating the expression of antigens that would normally be recognized by immune cells and by producing immunosuppressive factors that inhibit the function of immune cells. However, other studies have suggested that tumor-infiltrating immune cells can target and eliminate CSCs, thereby preventing tumor progression and recurrence [96–98]. Colorectal CSCs can evade detection by the innate immune system and form the TME through exosomes, cytokines and chemokines to create an immunosuppressive environment that facilitates cancer progression [99]. Enriched PD-L1 expression in CSCs contributes to cancer cell immune evasion. Hsu et al. showed that CSCs promote immune evasion by activating the EMT/β-catenin/STT3/PD-L1 signaling axis, but how CSCs communicate with cancer cells through this axis remains unclear [100]. In fact, this process is most likely mediated by exosomes. Another study showed that PD-L1-expressing exosomes can inhibit antitumor T cell responses (Fig. 3). In patients with melanoma, exosomal PD-L1 is also a marker of immune activation early after initiation of therapy with PD1-blocking antibodies and predicts a clinical response to PD1 blockade [101]. However, the mechanisms underpinning how CSC-derived exosomes regulate surrounding immune cells or contrast with non-CSC-derived exosomes remain elusive.

#### Exosomes facilitate information exchange of CSCs in the TME

As the importance of CSCs in tumor development and metastasis has been elucidated, special attention must be paid to their microenvironment because the tumor niche has a strong influence on tumor behavior. The TME includes CSCs and differentiated cancer cells, the extracellular matrix (ECM), MSCs, CAFs, immune cells, cytokines and growth factors. Exosomes are deeply involved in mediating information exchange between cells and the TME, modifying physiological and pathological processes by delivering specific molecules to their recipients [107]. Exosomes are also involved in the self-regulation and microenvironmental regulation of CSCs, maintaining the steady state of the TME, adapting and responding to changes in the TME [102, 108]. CSCs secrete various factors through exosomes to recruit and activate stromal cells, which reorganize the ECM, promote metastasis, drug resistance and tumor progression. Some factors such as IL-6, IL-8, IL-1β and VEGF, which are involved in the communication of CSCs with their environment, can be encapsulated in exosomes and freely released into the extracellular space [109–111]. Other ncRNAs secreted by CSCs could also be potent regulators involved in TME; glioma CSCs release exosomes carrying lncRNA MALAT1, which induces secretion of IL-6 and TNF-α from LPS-stimulated microglia cells. Oral squamous cell carcinoma (OSCC)-CSC-derived EVs transferred miR-134 and consequently promoted M2 macrophage polarization by targeting LAMC2 via the PI3K/AKT pathway in vitro and in vivo [112]. M2 macrophage-derived exosomal miR-31-5p can inhibit the tumor suppressor LATS2 gene and facilitate the progression of OSCC via inhibiting the Hippo signaling pathway [113]. Colorectal CSCs release miR-146a-loaded oncogenic exosomes to re-program non-CSCs and demonstrate the clinical relevance of exosomal miR-146a in predicting the TME in CRC patients [114]. Ovarian cancer stem-like cells activate the NF-κB and STAT3 signaling pathways through autocrine CCL5 signaling and mediate their own differentiation into endothelial cells [115]. On the other hand, other cells in the TME regulate CSCs via exosomes. Research has shown that CAF-derived exosomes can increase drug resistance to 5-fluorouracil in colon CSCs by activating Wnt signaling [116]. In addition, similar studies have shown that CAF-derived exosomes can activate STAT1 in breast cancer cells through the RIG-1 receptor. Subsequently, STAT1 activation further activates NOTCH3, which in turn increases the drug resistance of CSCs [117]. Studies have shown that myeloid-derived suppressor cells (MDSCs) can secrete the exosome S100A9, which enhances the activity of signal transducer and activator of STAT3/NF-κB signaling [118] and the production of prostaglandin E2 (PEG-E2) [119], promoting the stemness and survival of cervical cancer cells. Endothelial cells also induce NF-κB signaling in CSCs via secreted TNFα, creating a cytokine loop with immune cells and resulting in resistance to doxorubicin and cyclophosphamide [120]. This suggests that exosomes and their cargoes secreted by key components of the TME may have an impact on tumor progression.

#### CSC-derived exosomes enhance chemoresistance

CSCs are now considered to be a major cause of chemotherapy drug resistance. Existing studies have proposed various mechanisms of chemotherapy drug resistance of CSCs, such as drug outflows via ATP-binding cassette (ABC) transporter [121, 122], overactivation of DNA damage response, apoptosis avoidance, activation of survival pathways, cell cycle promotion, and/or changes in cell metabolism [123, 124]. CSC-derived exosomes can contribute to chemotherapeutic resistance by transferring miRNAs, proteins, and lipids to cancer cells, activating signaling pathways involved in cell survival and proliferation, inducing EMT, and modulating the TME [125]. It has been observed that the content of exosomes released by resistant cancer cells can cause sensitive cells to become resistant [126]. Exosomes from fibroblasts can induce the dedifferentiation of colorectal cancer cells into CSCs with stemness phenotype and function, thus enhancing the chemotherapy resistance of colorectal cancer [127, 128]. In addition, chemotherapy induces cancer cells to secrete exosomes containing drug-resistant miRNAs that act through the stem pathway, leading to increased stemness and drug resistance of CSCs [129–131]. Understanding such mechanisms is critical for developing effective therapies that can target CSC-derived exosomes and overcome chemotherapeutic resistance.

### Targeting CSC-derived exosomes in cancer progression

Given the crucial role of exosomes in cell communication, interfering with the interaction between CSCs and the TME by exosomes is an important direction for cancer therapy. Targeting CSC-derived exosomes to disrupt the signaling link between CSCs and TME or distant cells may provide a novel therapeutic strategy to impede the transmission of stemness characteristics and consequently thwart treatment resistance. Inhibition of exosome biogenesis is an increasingly promising approach for the treatment of cancer, with the potential to increase the efficacy of chemotherapy. Several studies have found that knockout of HRS, STAM1, and TSG101 can reduce exosome release and inhibition of these ESCRT components can alter vesicle properties and contents. In addition to the production of exosomes via an ESCRT-dependent pathway, sphingolipid ceramide also mediates the production of exosomes and hydrochloride hydrate (GW4869) can induce the inactivation of the acid sphingomyelinase. The Rab27 family is a class of small GTPase proteins that play an important regulatory role in the release of exosomes. Inhibition of Rab27a expression by RNAi can reduce the release of exosomes from cancer cells and inhibit the growth of tumors and the formation of metastatic clones [132, 133]. However, to date, there are no in vitro or in vivo research studies targeting CSC-derived exosomes for cancer treatment. Given that CSC-derived exosomes can reflect cellular content and carry specific markers, it is worth investigating the possibility of interfering with the synthesis or effect of exosomes by recognizing specific markers. On the other hand, studies have shown that the miRNA and protein content of CSC-derived exosomes present in body fluids of patients with liver, lung, prostate, and breast cancer are different from those found in normal human fluids [19, 95]. Although the proportion of CSCs in tumor tissues and the number of exosomes produced are small, considering the important role of CSCs in cancer progression and the "earlier" role of exosomes in metastasis and development, targeting exosomes from CSCs may also become an indicator for early diagnosis and prediction of metastasis for early intervention. Moreover, based on the specific markers of exosomes from CSCs, the niche of the primary tumor and the pre-metastatic niche can be identified [134, 135].

### Targeting CSCs with exosomal drug delivery

Since CSCs have unlimited proliferative potential that can drive tumorigenesis, the CSC theory may provide new insights into cancer therapy. In recent years, many efforts have been devoted to CSC-targeted therapies [136]. A number of promising new therapeutic strategies are being developed and have achieved good results [137–139], including CSC biomarker-mediated targeting, CSC mitochondrial targeting, CSC pathway targeting [140]. Nevertheless, the therapeutic benefits for practical use are still far from ideal due to the complicated microenvironment, special biological characteristics of CSCs, and drug delivery methods. For example, nanoparticles as a delivery vehicle deliver only 0.7% of the nanocarrier dose to solid tumors [141]. Given that multiple physiological barriers exist before reaching CSCs, how to ensure the uptake of anti-CSC agents by the target CSCs remains a prominent issue. Drug-loaded exosomes may serve as a next generation drug delivery mechanism that combines nanoparticle size with non-cytotoxic effects, a high drug carrying capacity, and a low immunogenic profile [142]. As exosomal carriers can provide advantages of both cell-based drug delivery and nanotechnology, interest in using exosomes for therapeutic approaches has exploded in recent years. Noteworthy, exosomes possess an intrinsic ability to cross biological barriers, including the most difficult to penetrate, the blood brain barrier (BBB) [143]. The combination of targeting molecules contained in exosomes, such as miR-21 and lncRNA UCA1, shows encouraging results for the treatment of certain aggressive cancers [144]. Kaori et al. used engineered biological nanoparticles analogous to exosomes to treat hepatocellular carcinoma by targeting liver CSCs [145]. Another team explored a potential way to use EVs as therapeutic agents to reprogram CSCs and stimulate their differentiation [146]. Other research using in vivo models has also explored the potential clinical applications of targeting exosomes. Tuying et al. developed biocompatible tumor cell-exocytosed exosome-biomimetic porous silicon nanoparticles (PSiNPs) as drug carriers for targeted cancer chemotherapy, and found that PSiNPs demonstrate significant cellular uptake and cytotoxicity in both bulk cancer cells and CSCs [147]. CSCs express specific surface markers, therefore, the binding between the biomarkers and the corresponding antibodies provides enhanced targeting through the permeability and retention effect, resulting in greater cellular uptake and higher drug concentration in CSCs and less damage to other cells [148]. Despite the advantages of CSC-derived exosomes for targeted drug delivery, there are several drawbacks. First, the size of exosomes is relatively small, which limits their ability to carry large therapeutic molecules. Second, exosomes are expensive and time-consuming to produce, making them costly for therapeutic use. Finally, the TME of the target cells can reduce the stability of the cargo inside the exosomes. These limitations can reduce the efficacy of the drug, resulting in suboptimal therapeutic outcomes. For example, despite their unique lipid and protein composition, exosomes have a very short half-life and are rapidly cleared from the circulation after in vivo administration, with less than 5% of the injected dose of exosomes remaining in the circulation after 3 h [149].

### Current challenges and perspectives

In summary, CSCs are highly tumorigenic, significantly resistant to traditional cancer therapies, and a cause of local tumor recurrence and distal metastasis. Selective targeting of CSCs is a promising therapeutic strategy to eliminate the development of human cancer and reduce the risk of recurrence [150]. To date, only a few studies have focused on CSC-derived exosomes. However, a growing body of evidence has confirmed that exosomes from complex tumor tissue samples are composed of multiple cell subsets and have potent immunomodulatory properties that promote tumor progression. Clearly, it is important to distinguish whether these exosomes are derived from CSCs or simply from non-CSCs to determine the underlying mechanisms, which may provide promising options in the search for novel diagnostic and therapeutic approaches targeting CSCs. For example, further study of CSC-derived exosomes and their effects on the immune system may reveal previously undiscovered mechanisms that inhibit anti-tumor immunity and thereby shed light on new therapeutic targets for drug immunotherapy. Some of the mechanisms, including exosomes, that allow CSCs to evade the immune system are also being targeted in immunotherapy approaches that aim to activate the immune system and specifically target cancer cells.

Despite this potential, it remains technically challenging to effectively isolate CSCs and their exosomes. First, studies performed only in vitro or in immunodeficient mouse models cannot simulate the complex immune characteristics of CSCs in vivo. In addition, the small number of CSCs makes in vivo immune mouse experiments very challenging. CSCs require a specific niche for survival in vivo, and most current studies use isolated CSCs that lack the TME, making it difficult to define the relationships between different cell types [151]. Second, because CSCs share some signaling pathways with normal stem cells, not all regulatory factors that contribute to CSCs are suitable as therapeutic targets for cancer therapy, and some targets that have proven useful so far may be unusable in application. How to make specific exosomes target and bind to CSCs without causing potential damage to other stem cells or somatic cells has always been a challenge in this field, and new methods for developing exosomes that target the microenvironment of CSCs are very promising.

## Data Availability

Not applicable.

## Abbreviations

ABC: ATP-binding cassette
BBB: Blood brain barrier
CAFs: Cancer associate fibroblasts
CSCs: Cancer stem cells
ECs: Endothelial cells
ECM: Extracellular matrix
EMT: Epithelial-mesenchymal transition
ESCC: Esophageal squamous cell carcinoma
ESCRT: Endosomal sorting complexes required for transport
ESE: Early sorting endosome
EVs: Extracellular vesicles
FACS: Fluorescence-activated cell sorting
ILVs: Intraluminal vesicles
MACS: Magnetic-activated cell sorting
MDSC: Myeloid-derived suppressor cells
miRNAs: MicroRNAs
MSCs: Mesenchymal stem cells
MVBs: Multivesicular bodies
ncRNA: non-coding RNA
OSCC: Oral squamous cell carcinoma
PMN: Pre-metastatic niche
TME: Tumor microenvironment
SP: Side population
SFM: Serum-free medium
